# Multiple cavernous hemangiomas in the orbit: A case report and review of the literature

**DOI:** 10.1097/MD.0000000000020670

**Published:** 2020-07-17

**Authors:** Chaohua Deng, Weikun Hu

**Affiliations:** Department of Ophthalmology, Tongji Hospital, Tongji Medical College, Huazhong University of Science and Technology, Wuhan, China.

**Keywords:** cavernous hemangioma, multiple, orbital tumors, visual impairment

## Abstract

**Rational:**

Cavernous hemangiomas are one of the most common benign primary orbital lesions. These tumors are insidious in onset, slowly progressive and present more often in middle aged women. Multiple orbital cavernous hemangiomas are extremely rare, and only a few cases have been reported in the published literature.

**Patient concerns:**

Here, we report the diagnosis and treatment of multiple cavernous hemangiomas in the right orbit of a female patient with impaired visual acuity and proptosis of the eye for more than 10 years.

**Diagnosis:**

Magnetic resonance imaging of the orbit showed a giant and irregular soft mass filling the intraconal and extraconal space of the right orbit, compressing the right optic nerve. After tumor resection, histopathological examination confirmed the diagnosis of cavernous hemangioma.

**Interventions:**

A lateral orbitotomy was performed and a total of 13 tumors were excised, with the largest tumor measuring approximately 2.5 × 3.0 cm.

**Outcomes:**

The visual acuity of the patient was preserved, with only a slightly dilated pupil of the right eye. The follow-up period was 6 months with no signs of recurrence.

**Lessons:**

Multiple cavernous hemangiomas in the orbit is rare and should be excised surgically as soon as possible.

## 1 Introduction

Cavernous hemangioma is the most common benign orbital tumor in adults, which includes approximately 10% of orbital tumors and occurs often in the intraconal space as well-circumscribed lesions.^[1]^ Patients present most commonly with slowly progressive painless proptosis and vision impairment to complete blindness if the lesions are located at a deeper position or have damaged the surrounding tissues, such as the optic nerve.^[2]^ Radiological examinations, such as computerized tomography (CT) and magnetic resonance imaging, have a fundamental role in its diagnosis. Most of the tumors are unilateral or solitary, and multiple or bilateral lesions within the orbit are rarely reported.^[3,4]^ Here, we report a case of multiple cavernous hemangiomas in the right orbit of a middle-aged woman who had a history of proptosis for more than a decade and experienced an acute progression within 6 months.

## 2 Case report

**Figure 1:**
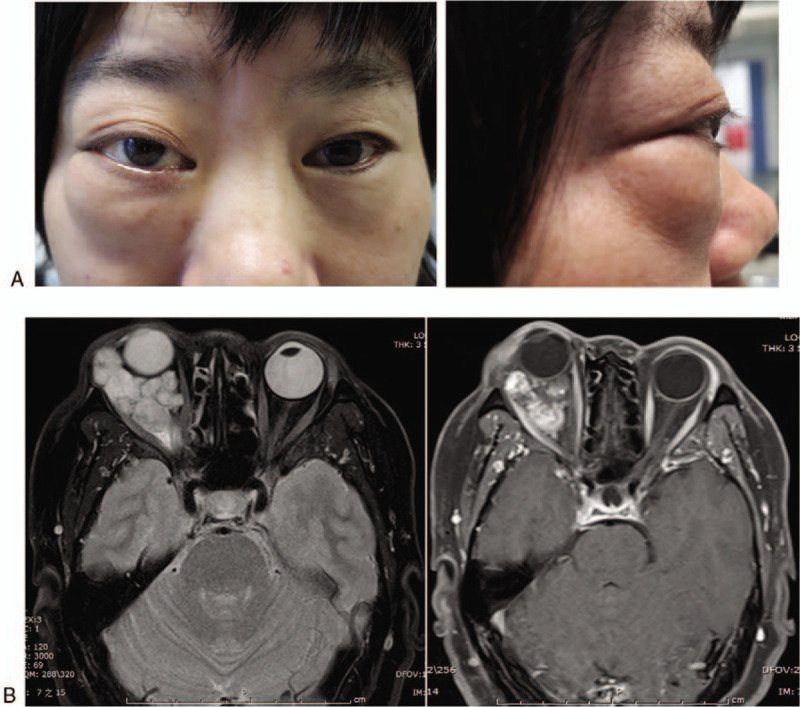
Ocular appearance and radiological examinations of the patient. (A) Front and lateral view of the patient. (B) T2-weighted magnetic resonance imaging showed a giant and irregular soft mass filling the right orbit up to the subcutaneous layer around the orbit, compressing the right optic nerve. T1-weighted image after gadolinium injection showed the heterogeneous and multi-focal enhancement signals in the masses.

**Figure 2:**
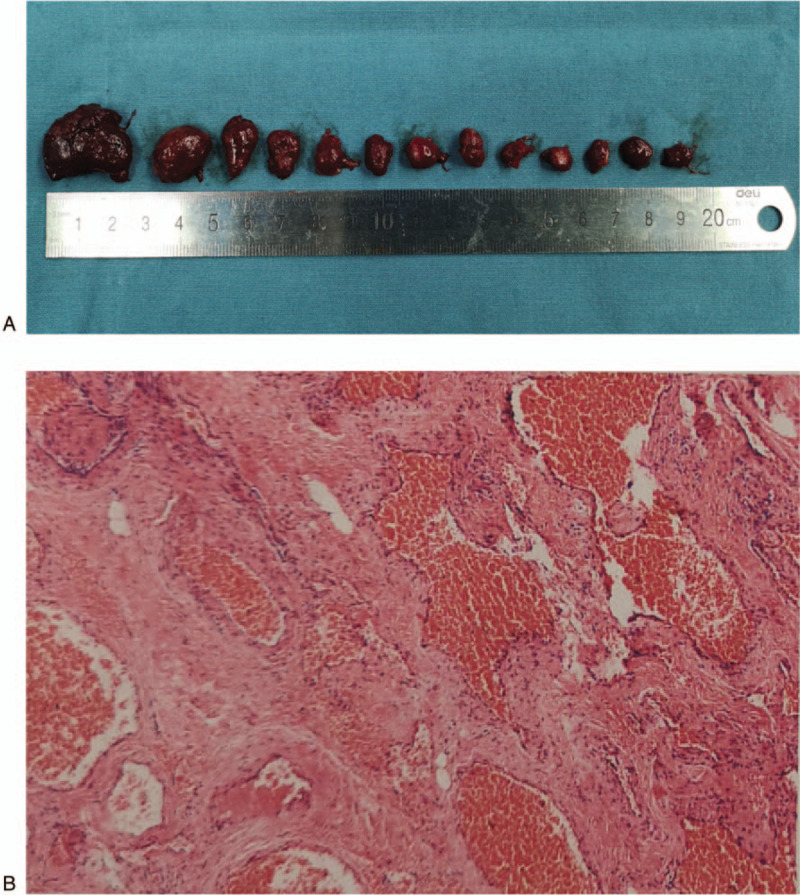
Illustration of tumor specimen and pathological examination results after the surgery. (A) A total of 13 tumors were excised which had a complete capsule and smooth surface, and were purplish red and of varying size. The largest tumor was measured approximately 2.5 × 3.0 cm. (B) Histopathological examination reveals the endothelium-lined and blood-filled lesions which are surrounded by abundant smooth muscle cells and fibrous tissues. (Hematoxylin & eosin stain, original magnification, × 40).

A 47-year-old female patient was admitted to the hospital with a complaint of painless progressive proptosis of the right eye for more than 10 years and a significant decrease in visual acuity for 6 months. On physical examination, the best-corrected visual acuity (BCVA) for the right eye was 3/10 and 10/10 for the left eye. There was 10 mm of right axial proptosis. The upper eyelid of the right eye had mild prolapse, covering the upper corneal margin by approximately 3 mm. A 3.0 × 3.0 cm soft mass was visualized and palpated in the lower eyelid (Fig. 1A). The eye movements were restricted during ophthalmic examination. Both the eyes had normal intraocular pressure without obvious abnormalities on anterior segment examination. Magnetic resonance imaging of the orbit showed a giant, irregular soft mass filling the intraconal and extraconal space of the right orbit up to the subcutaneous layer around the orbit, compressing the right optic nerve. The lesion was isointense on T1-weighted images and hyperintense on T2-weighted images with gradual multi-focal enhancement after gadolinium injection (Fig. 1B). These findings were compatible with the diagnosis of orbital giant cavernous hemangioma. A lateral orbitotomy was performed and the tumors were excised during the operation. All of the tumors had a complete capsule and smooth surface, and were purplish red and of varying sizes. A total of 13 tumors were excised, with the largest tumor measuring approximately 2.5 × 3.0 cm (Fig. 2A). Histopathological examination confirmed the diagnosis of cavernous hemangioma (Fig. 2B). In the postoperative period, no complications were observed and vision was preserved (BCVA, 2/10), with only a slightly dilated pupil in the right eye. Until 6 months of follow-up, there was no sign of recurrence.

## 3 Discussion

Cavernous hemangiomas are the most common benign primary tumor of the orbit, which are vascular malformations consisting of dilated blood vessels separated by fibrous tissue and most frequently found in middle-aged women.^[1]^ In the present study, the patient diagnosed with orbital cavernous hemangioma had multiple tumors within the same orbit, with a total of 13 tumors of different sizes, which is seen very rarely in clinical practice.

To our knowledge, only 15 cases of multiple cavernous hemangiomas occurring within the same orbit have been reported (summarized in Table 1).^[2,3,5–17]^ Among those cases, one was that of bilateral intraorbital hemangiomas with two tumors in the right orbit and three tumors in the left orbit. Twelve patients were female, and three were male. The mean age was 41.9 years, ranging from 7 to 63 years. The right orbit was involved in 10 patients, and left orbit was involved in 5 patients. The average number of tumors excised with surgery was 5.3, with a maximum 15 tumors in 2 cases. There were 3 cases with recurrence of the tumor during the follow-up period: the first case reported 15 years after the complete excision of the tumors; the second case recurred 8 years after surgery. The third case took 6 year for the first recurrence and 24 years for the second relapse.

**Table 1:**
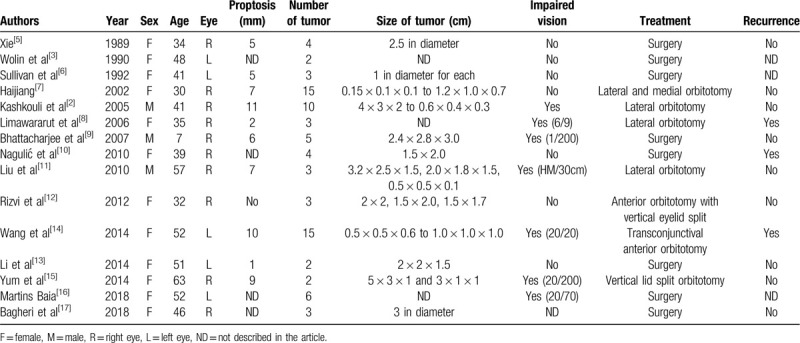
Clinical features of summarized literature of cases reporting multiple cavernous hemangiomas in the orbit.

In large case series, there were no reports of tumor recurrence if the lesions were excised completely. However, if the tumors were incompletely excised, recurrence may occur. Yan and Wu^[18]^ reported that in 9 incomplete excised cases, 3 cases experienced recurrence. Orbital cavernous hemangioma expresses tumor growth factor bFGF, which can stimulate the growth of endothelial cells and vascular smooth muscle cells and may participate in the growth of tumor.^[19]^ Even after complete excision of the tumor, the secreted and remaining bFGF may still have the potential to stimulate the growth of cavernous hemangioma. In addition, the progesterone receptor is present in the orbital cavernous hemangioma,^[20]^ implying a hormone-dependent mechanism in tumor growth. Undeniably, we cannot exclude the remnants or small hidden tumors within the orbit, even after complete excision. The general therapeutic outcomes of the surgery were excellent for the majority of reported cases, and the recurrence rate was very low.

Multiple cavernous lesions can also present in a bilateral form. To date, 13 cases of bilateral cavernous hemangiomas have been reported.^[4]^ Interestingly, in all the reported cases, the symptoms were unilateral involving only the orbit harboring the larger mass. However, surgical management is the same option as for unilateral cavernous hemangioma and should be limited to symptomatic lesions.

These patients usually had a long course of disease, and the tumor filled the orbital cavity, which compressed the optic nerve and extraocular muscle, resulting in irreversible visual impairment, suggesting that such patients should undergo surgical excision as soon as possible.

Although extremely rare, cavernous hemangiomas should be considered in the differential diagnosis of patients with multiple orbital mass lesions. In the presence of multiple cavernous hemangiomas, systemic evaluation of the patients should be done to rule out multi-centric lesions. The tumors should be completely excised by surgery and a very long follow-up of these patients is recommended to monitor recurrence.

## Acknowledgments

We thank the patient for granting permission to publish this information.

## Author contributions

**Conceptualization:** Chaohua Deng, Weikun Hu.

**Resources:** Weikun Hu.

**Supervision:** Weikun Hu.

**Writing – original draft:** Chaohua Deng, Weikun Hu.

**Writing – reviewing & editing:** Chaohua Deng, Weikun Hu.
